# Cord Blood-Derived Hematopoietic Stem/Progenitor Cells: Current Challenges in Engraftment, Infection, and *Ex Vivo* Expansion

**DOI:** 10.4061/2011/276193

**Published:** 2011-04-27

**Authors:** Katsuhiro Kita, Jong O. Lee, Celeste C. Finnerty, David N. Herndon

**Affiliations:** ^1^Department of Surgery, Shriners Hospitals for Children, University of Texas Medical Branch, 815 Market Street, Galveston, TX 77550, USA; ^2^Institute for Translational Sciences, University of Texas Medical Branch, 815 Market Street, Galveston, TX 77550, USA; ^3^Sealy Center for Molecular Medicine, University of Texas Medical Branch, 815 Market Street, Galveston, TX 77550, USA; ^4^Department of Pediatrics, University of Texas Medical Branch, 815 Market Street, Galveston, TX 77550, USA

## Abstract

Umbilical cord blood has served as an alternative to bone marrow for hematopoietic transplantation since the late 1980s. Numerous clinical studies have proven the efficacy of umbilical cord blood. Moreover, the possible immaturity of cells in umbilical cord blood gives more options to recipients with HLA mismatch and allows for the use of umbilical cord blood from unrelated donors. However, morbidity and mortality rates associated with hematopoietic malignancies still remain relatively high, even after cord blood transplantation. Infections and relapse are the major causes of death after cord blood transplantation in patients with hematopoietic diseases. Recently, new strategies have been introduced to improve these major problems. Establishing better protocols for simple isolation of primitive cells and *ex vivo* expansion will also be very important. In this short review, we discuss several recent promising findings related to the technical improvement of cord blood transplantation.

## 1. Umbilical Cord Blood (UCB) as a Promising Source of Hematopoietic Cells for Transplantation

Bone marrow (BM) has been used for years to treat hematopoietic disorders such as leukemia. Indeed, the first BM infusion was performed in 1957 [1]. Although multiple clinical studies with large numbers of patients have greatly advanced our knowledge of BM transplantation (e.g., [2–4]), this approach still faces three major obstacles. (1) Finding the desired donor, such as a human leukocyte antigen- (HLA-) identical sibling, is often difficult. A closely HLA-matched, unrelated donor is an alternative option, though the mortality associated with this type of transplant is high. (2) Graft-versus-host disease (GVHD) occurs in recipients at high frequencies, in some cases reaching 80–90% [3]. Transplantation of BM from an unrelated donor is an option. However, this is risky, and the mortality rate (estimated as 30–50% [4]) is higher than that seen in HLA-matched cases. For the treatment of hematopoietic malignancy, autografts are not available. (3) Isolation of BM requires invasive procedures, and mortality from infection can be as high as 50%.

BM is not the sole source of hematopoietic cells for transplantation. Since the late 1980s, researchers have investigated the feasibility of using umbilical cord blood (UCB) transplantation as an alternative to BM transplantation. The first hematopoietic reconstruction using UCB was reported in 1989 [5]. Transplantation of HLA-identical donor-derived UCB successfully reconstituted a patient suffering from Fanconi's anemia [5]. Subsequent studies showed that UCB or placental blood (hereafter also referred to as “UCB,” because it shares a similar origin as UCB) is a useful alternative to BM [6–8]. Initially, UCB was mainly used in children. However, in 2001, a large multicenter trial suggested that UCB is still effective for hematopoietic engraftment in adults [9]. Although hematopoietic reconstruction with UCB seems to be more difficult in adult patients due to age-related complications [8, 10] and the dose of cells required, this trial showed that UCB from unrelated donors or HLA-mismatched donors still reconstituted hematopoietic systems. This finding increased the popularity of using UCB as a source of blood cells for transplantation. This review will begin with a discussion of hematopoietic stem cells (HSCs)/hematopoietic progenitor cells (HPCs). This will be followed by a discussion of how transplants can be improved through advances in cell culture/bioengineering. Large-scale culture systems and further improvements in engraftment will be particularly important for clinical application of UCB transplantation.

## 2. Discovery and Enrichment of HSCs/HPCs

The discovery that stem cells (SCs) are present in human UCB was made by Meulen's group in 1978 [11]. Similar reports by the other groups followed [12, 13]. Meulen's group reported that the number of SCs noticeably decreased in 8- to 10-day-old infants [11], a finding that was confirmed by another group shortly thereafter [14]. This suggested that a dramatic reduction of HSCs occurs in UCB upon aging. Thus, UCB cells should be processed and stored as early as possible.

Multipotent cells from either BM or UCB can produce several different hematopoietic cell colonies when grown in agar. Kirshner and Goldberg compared the cells produced by three different sources of stem/progenitor cells (BM, peripheral blood, and UCB) for the first time in 1980 [12] and noted differences in the cells produced by UCB cells. Two striking differences existed between BM- and UCB-derived colonies: (1) UCB produced a lower percentage of eosinophils (approximately 2-3 fold less) as well as (2) more macrophage and granulocyte colonies [12]. Characterization and comparison of stem/progenitor cells in BM and UCB still continues, and completely new trends might appear. However, this early study clearly pointed to major differences between BM and UCB.

Weismann's group was the first to isolate and purify mouse HSCs from BM [15]. By this time, studies had already suggested the presence of cells capable of repopulating all hematopoietic lineages. Weismann's group purified a Thy-1^low^Lin^−^Sca-1^+^ fraction of HSCs. Later, Rhodamine 123-based staining revealed that Lin^−^Sca^+^ cells are heterogeneous [16], suggesting that the initial criterion may not be sufficient to purify the most primitive HSCs. Because HSCs or primitive HPCs constitute only a very small fraction of total UCB, identifying primitive HSCs/HPCs for *ex vivo* expansion is very important. In the early 1990s, CD34 was already known to be a useful cell surface antigen for enriching HSCs (reviewed in [17]). Reams of studies have addressed the use of additional markers, and some have uncovered discrepancies suggesting that CD34^+^ may not be necessary (see further discussion in later paragraphs of this section) [18–20]. Nevertheless, CD34^+^CD38^−^ is a widely accepted criterion for enriching primitive HSCs/HPCs [21]. c-Kit (stem cell factor receptor) [22, 23] and Thy-1/CD90 [24] have also been proposed as cell surface markers of primitive HPCs, though they are not well accepted. CD34^+^CD38^−^, Lin^−^, and probably Sca^+^ may be reasonable criteria for selecting a highly enriched, primitive fraction from UCB. In a recent study, CD150^−^CD48^−^Lin^−^ was used to enrich HSCs from mouse peripheral blood cells [25]. New studies may use better, more updated criteria for enriching the HSC/HPC fraction from UCB cells; however, whether these selection parameters are useful for identifying the most primitive HSCs in human UCB or other sources is unclear. Broxmeyer's review provides a useful guide to the current standard protocol for isolation of HSCs/HPCs from UCB [26].

As already mentioned, controversy exists over whether CD34 expression is reliable for selection of HSCs. CD34^−^ and CD34^+^ cells may be interchangeable phenotypes (reviewed in [27]). Discrepancies in cell surface expression of CD34 may be explained by the hypothesis that CD34 is mainly expressed in intracellular compartments at early stages. BM CD34^−^ cells were reported to generate CD34^+^ cells after transplantation. This may be supported by the idea that CD34^low/-^ cells home toward BM, where the cells appear to gain cell surface expression of CD34 [20]. Although several papers described potentially primitive CD34^−^ cells [18–20], whether these cells express CD34 in intracellular compartments is unknown [28]. The above uncertainty might preclude the CD34^+^ phenotype from being used as the gold standard in preparing primitive cells for clinical application.

In current HSC/HPC research, several antibodies are combined to select primitive cells. This provides very useful information for laboratory-level studies. However, as in some examples discussed above, cells could acquire or lose phenotypes during *ex vivo* culture. For this reason, other methods may be more convenient in clinical settings. The use of chemical or enzymatic activity-based selection would be a great advantage if available and would minimize the use of antibodies, lowering production costs. Interestingly, HPCs have been shown to highly express cytosolic aldehyde dehydrogenase (ALDH) [29]. Storms et al. successfully synthesized a fluorochrome- (BODIPY-) conjugated substrate for ALDH, which produces fluorescence upon enzymatic cleavage by ALDH [30]. Using this novel tool, they were able to perform a relatively simple, fluorescent dye-based selection of HPCs.

Exploring the best set of markers to isolate the cells harboring the best potential for transplantation is very important. However, using a combination of several cell surface antigens for the selection of HSCs/HPCs will considerably reduce the number of cells in the final fraction. Pursuing further markers to obtain absolutely pure HSC/HPC fractions may not be necessary. Simple, CD34-positive selection still yields cells with enough expansion capacity [31]. In addition, it should be noted that “primitive” cells and “proliferative” cells do not necessarily share the same characteristics, and the most important goal may be to secure sufficient capacity for expansion and *in vivo* engraftment.

## 3. Transplantation

### 3.1. Immaturity of UCB Cells

Because BM transplantation has a longer history than UCB transplantation, fundamental questions remain about UCB transplantation. For example, “what is special in UCB and what advantages does UCB (HSCs/HPCs) have?” Clinical trials have basically shown that UCB transplantation causes less GVHD and graft failure [6–9, 32, 33]. Recipients seem to be less sensitive to HLA-mismatching when UCB is used as a source of HSCs/HPCs.

One clinically interesting fact is that UCB myeloid progenitor cells are relatively chemoresistant [34]. This is very important because blood cell transplantation is often combined with intensive chemotherapies to reconstruct host immune systems. Use of chemoresistant UCB cells with more intensive chemotherapies may avoid the relapse of diseases that are sometimes observed after the treatment of hematopoietic malignancy.

If UCB cells have better potential than BM cells to be engrafted into recipients, then what is the underlying mechanism(s)? The immunological potential of T cells is thought to be one of the key determinants of successful long-term engraftment of transplanted HSCs. A study by Harris et al., which was the first to systematically measure a significant number of samples, showed that UCB cells were unresponsive compared to peripheral blood cells, although IL-2 stimulation could still induce NK cell-mediated lysis of target cells [35]. A slightly lower level of T cell receptor expression was also reported. The authors found that UCB contained CD3^−^ cells, which were not present in peripheral blood [35]. In addition, although the percentage of B cells was comparable between peripheral blood and UCB, approximately 50% of B cells in UCB were CD5^+^, a marker of immature B cells [35]. This finding led the authors to suggest that UCB lymphocytes are functionally immature. UCB cells are less likely to cause GVHD than BM or peripheral blood cells. Interestingly, UCB also showed less cytotoxicity against NK cell-resistant tumor cells. This finding requires careful investigation, as less antitumor activity in reconstituted hematopoietic cells could increase the risk of relapse of hematopoietic malignancies. That is, functional immaturity of UCB lymphocytes may be a “double-edged sword.” UCB transplantation apparently yields better responses in recipients, such as decreased GVHD; however, the long-term clinical outcomes of using UCB may not be significantly better than those achieved with other blood sources.

Although T-cell depletion does not seem to be necessary for UCB transplantation [36], subpopulations of patients undergoing UCB transplantation still experience problems such as GVHD and a relatively poor survival rate, possibly due to the higher percentage of HSCs or immaturity of hematopoietic cells in UCB [8, 10]. A small dose of transplanted cells is also thought to be associated with graft rejection. Progenitor cell content may be particularly important for successful engraftment [37]. Potential solutions are discussed in Section 4.

### 3.2. Animal Studies of UCB Engraftment and Repopulating Potential

A comparison of UCB, BM, and peripheral blood cells has shown that UCB cells engraft better than cells from the latter two origins when transplanted into nonobese/severe combined immunodeficient (NOD/SCID) mice. *In vitro* and *in vivo* studies conducted by Kim et al. revealed that UCB-derived CD34^+^ cells not only have better colony-forming ability than BM-derived CD34^+^ cells, but also show significantly better engraftment in NOD/SCID mice [38]. One very interesting finding of this study is that the level of chimerism is better in mice transplanted with total UCB cells than partially purified UCB cells (CD34^+^ cell fraction) [38]. This finding implies that the CD34^−^ fraction may contain some cells that support the engraftment of transplanted cells. If this finding is applicable to humans, again, enrichment of cells based on cell surface CD34 may not be necessary for transplantation. Interestingly, UCB is thought to contain a significantly higher number of multipotent and CD34^+^CD38^−^ cells than BM. Hoffman's group developed an *in vivo* competition assay to analyze the BM repopulating potential of UCB cells. They established mice implanted with human fetal bones [39]. After irradiation, HLA-mismatched UCB or adult BM cells were injected individually or in combination. UCB and adult BM CD34^+^ cells showed similar multilineage hematopoiesis when equal numbers of UCB or BM cells were injected individually. However, UCB outcompeted BM cells in repopulating BM when both were simultaneously injected [39]. This result suggests UCB HSCs/HPCs have superior capacity to engraft and repopulate. Thus, studies in animal model systems support the idea that UCB is superior source of hematopoietic cells than BM.

## 4. Approaches to Improving UCB Transplantation Outcomes

### 4.1. Engraftment

Aside from increasing the dose of cells, accelerating the homing of HSCs/HPCs to hematopoietic tissues could considerably improve engraftment. Although the exact molecular mechanisms underlying HSC/HPC homing remain unclear, a chemokine, stroma-derived factor-1*α* (SDF-1*α*), has been shown to play an important role in BM engraftment of HSCs [40]. *In vitro* experiments showed that this factor induced chemotaxis of UCB progenitors as well as BM cells [41]. In spite of this *in vitro* chemotactic activity, clinical trials have shown that neutrophil recovery is slower after UCB transplantation than after BM transplantation [32, 36], a finding that might reflect slower migration of UCB cells than BM cells toward the BM after transplantation. The functional immaturity of UCB cells may make it more difficult for these cells to rapidly home to the correct location (i.e., BM). This might explain why UCB transplantation requires a longer time to show beneficial effects. Interestingly, the cell surface dipeptidylpeptidase, CD26, is suggested to negatively regulate *in vivo* homing and engraftment of HSCs [42]. This protease can cleave the N-terminal dipeptide from several cytokines, including a chemokine SDF-1 (CXCL12) [43]. This proteolysis dampens the biological activity of SDF-1 in chemotaxis [44]. Moreover, a recent study by the same group demonstrated that the percentage of CD26^+^ cells is higher in immature, primitive cells (CD34^+^CD38^−^) than in mature cells [45]. These findings suggest that inhibition of CD26 may be a promising strategy for improving clinical outcomes. Enhancing HSC/HPC homing may reduce the dose of cells required for transplantation.

### 4.2. Infection and Relapse

Infection is the major complication following transplantation. In one study, all patients experienced at least one infection after transplantation [46]. One characteristic of UCB transplantation is the slow neutrophil recovery that occurs relative to BM transplantation [36]. Although increasing the dose of transfused cells certainly shortens the neutrophil recovery time [8], this may not be a realistic solution because of the low availability of UCB cells. Unfortunately, treatment of patients with granulocyte-colony stimulating factor or granulocyte macrophage-colony stimulating factor is not effective in improving neutrophil recovery [32]. It is not yet clear whether slower neutrophil recovery increases morbidity and mortality by infection. Nevertheless, cotransplantation of neutrophils or their precursors might improve clinical outcomes. Indeed, in one study, such cotransplantation accelerated neutrophil recovery and reduced bacterial and fungal infections [47]. Because the number of neutrophils is limited in blood samples, *ex vivo* expansion of neutrophils is necessary. Few studies have tackled this topic. The most recent, conducted by Timmins and colleagues, has yielded very encouraging results with regard to clinical application of neutrophil coinfusion. The authors successfully expanded functional neutrophils in a large-scale bioreactor at a volume up to 10 L [48]. Expanded neutrophils showed respiratory burst activity to generate reactive oxygen species and killed the fungus, *Candida albicans*. The system that the authors used is capable of processing culture volumes up to 500 L [48].

Most recently, a new strategy has been introduced to combat infections and leukemia relapse, major factors causing death of transplant recipients. Michlethwaite and colleagues have generated cytotoxic T lymphocytes (CTLs) harboring both antiviral and antileukemic activities [49]. A retrovirus-coded single-chain anti-CD19 fragment was introduced into T cells prepared from peripheral blood and UCB. The resulting CTLs showed specific and strong cytolytic activity against B-cell acute lymphoblastic leukemia as well as against cell lines, demonstrating its clinical potential in combating leukemia relapse and viral infections concomitant with blood transplantation. Although the introduction of a retrovirus-encoded chimeric gene may bring some concerns, this strategy is very straightforward, and the outcome of the study suggests that this approach holds promise in reducing infections and leukemia relapse-related mortality in patients in the future.

## 5. Expansion

### 5.1. Culture Supplements Used for Expansion

The limited number of HSCs/HPCs is the major obstacle in blood transplantation. Until the number of transplantable cells can be increased to amounts necessary for adult patients, the application of UCB should be limited to pediatric patients. Efficient methods are needed to obtain a larger quantity of primitive cells from UCB. For this reason, developing simple and realistic protocols for industrial-scale expansion of primitive cells in UCB is essential.

Usually, the use of animal products such as fetal bovine serum is inevitable for the propagation of SCs. Since the HSC field is well established, a serum-free culture system is available to expand HSCs/HPCs, though room for improvement still exists. A combination of several cytokines is typically used for *ex vivo* culture of HSCs/HPCs. Although the exact combination differs among laboratories, it usually consists of several cytokines such as Flt3/Flk2 ligand [50, 51], stem cell factor (SCF) [52, 53], interleukin-3 (IL-3) [54–56], IL-6 [57], and thrombopoietin [58–60]. Among these cytokines, SCF probably has the longest history as a supplement. Flt3/Flk2 ligand may be more effective than SCF [61]. Flt3 ligand knockout mice exhibit serious problems in the early development of HPCs [62], suggesting that including Flt3/Flk2 ligand in the media is essential. Interestingly, gp130 signaling (through IL-6) was reported to synergistically enhance the effect of SCF [63]. The same trend was also reported for Flt3 ligand [64]. Thus, gp130 signaling appears to be supportive. It should be noted that IL-6 alone was not shown to be effective [54, 63, 64]. The effect of IL-3 may need to be carefully evaluated, because the addition of IL-3 was reported to suppress the number of colony-forming cells [65]. It also reduced the reconstituting activity of HSCs [65]. Other cytokines, such as IL-7 and IL-11, are also suggested to promote proliferation of HSCs/HPCs. IL-7 is likely more effective in promoting T-cell expansion [66]. Thus, it might not be suitable for expanding primitive HSC/HPC populations. An unbalanced increase in T-cell populations in *ex vivo* culture may increase the risk of graft rejection. IL-11 might not be essential, since the absence of IL-11 did not result in a dramatic difference after 10 weeks of culture [67]. Although some suppliers can offer bulk recombinant cytokines in these days, the use of cytokines still increases the cost of expansion. Using a combination of several cytokines also makes it difficult to know the exact molecular mechanism facilitating the expansion of HSCs/HPCs. Further evidence suggests that a feeder layer-based coculture system may also be good for *ex vivo* culture. For example, porcine microvascular endothelial cells (PMVECs) have been reported to be effective for *ex vivo* expansion of UCB cells [68]. Among several studies investigating different types of feeder layer-based culture systems, that by Rosler et al. showed that the PMVEC coculture system was 5-fold and 241-fold more effective than a stroma-free culture system (supplemented with cytokines) for expansion of CD34^+^ and CD34^+^CD38^−^ cells, respectively (Figure 2 in [39]). However, coculture systems are probably not suitable for large-scale production of cells. New culture methods or supplements should provide more advantages, while remaining simple. Two recent lines of studies, discussed below, may bring important breakthroughs in the *ex vivo* expansion of HSCs/HPCs.

Notch signaling has been proposed as a target to promote efficient expansion of HSCs. The human homolog of *Drosophila* Notch was originally discovered as a gene highly expressed in CD34^+^ hematopoietic cells [69]. Incubation of immobilized Notch ligand dramatically increases (by approximately 100-fold) the number of CD34^+^ cells and enhances repopulating ability in NOD/SCID mice [70]. Although Notch signaling was shown to initiate lymphoid differentiation and enhance self-renewal, a repopulation assay using NOD/SCID mice clearly demonstrated that Notch ligand-incubated cells have far greater ability to reconstitute host marrow. Notch ligand-treated cells injected into mice were also capable of thymic engraftment, which is typically unsuccessful [70]. Recently, the same group has provided encouraging data, including preliminary results of a phase 1 trial showing that CD34^+^ UCB progenitors expanded with Notch ligand engraft better than control cells [71]. One of the important observations in this study is that transplantation of Notch ligand-expanded cells substantially shortens neutrophil recovery time [71], a feature thought to predict successful engraftment. Because clinical trials are ongoing, Notch ligand-mediated *ex vivo* expansion may alter clinical regimens soon. One potential pitfall relates to the fact that the successful results of this study were obtained using an immobilized Delta1 fusion protein. It should be noted that Delta1-mediated Notch activation can induce apoptosis of cells under certain conditions (such as when there is a high density of ligand) [72]. The development of a more effective Delta1 ligand with reduced cytotoxicity would be desirable for Notch ligand-mediated large-scale expansion.

Biological compounds such as growth factors/cytokines may not be the only compounds that can be used to promote expansion. A recent study has shown that a chemical compound could also be used for this purpose [73]. Chemical compounds would be superior to biological compounds because of lower production costs and greater ease of quality control. Microscope-based high-throughput screening has identified aryl hydrocarbon receptor antagonists as potential drug candidates for promoting *ex vivo* expansion of HSCs [73]. Currently, these compounds require a cytokine cocktail. However, further development of this finding may eventually lead to chemically defined culture media for clinical-scale production of HSCs. Table 1 shows currently available supplements (including those for feeder cell-based cultures) for *ex vivo* expansion of HSCs/HPCs and shortly summarizes the advantages and disadvantages of each.

### 5.2. Large-Scale Culture Systems

Conventional culture flasks and gas-permeable blood bags [31] are the most widely used systems for the expansion of hematopoietic cells. The first automated bioreactor culture system for BM HPCs was described in 1993 [74]. This first system used a continuous perfusion device, which was connected to a syringe pump to allow fresh cell culture medium to be supplied. This system, with supplements, allowed for up to a 30-fold expansion of granulocyte-macrophage progenitor cells; however, it was less effective for other progenitors [74]. Advantages/disadvantages and other factors (e.g., oxygen, pH, shear stress) are well summarized in Nielsen's review [75]. Recently, a rotating wall vessel was used for *ex vivo* expansion of UCB cells [76]. Although the total cell number was increased by 435 times, the expansion of CD34^+^ cells was approximately 30-fold [76]. Since a simple Teflon cell culture bag yields an average expansion of 113-fold in CD34^+^ cells [31], further improvements should be explored for more efficient large-scale culture.

## 6. Other Potential Applications of UCB Cells

Aside from being used for the treatment of hematopoietic malignancy, SC transplantation has been used to improve recovery from injuries and to accelerate wound healing. These transplantations have been conducted with mesenchymal stem cells (MSCs). However, the heterogeneity of UCB cells (a mixed system of endothelial progenitors, MSCs, and HSCs/HPCs) may be useful for the systemic treatment of conditions such as traumatic injury. To our knowledge, no solid evidence shows that cord blood HSCs/HPCs are efficacious in wound healing. Nevertheless, one case report using CD34^+^ cord blood cells suggests that the application of cord blood SCs is of clinical interest [77]. The authors also showed that cord blood cells can be mixed with fibrin gels, which is a popular material to apply cells onto wounds in clinical settings. Further studies will be necessary to see if cord blood SCs are effective for wound healing. Whether nonmesenchymal cells in cord blood (i.e., HSCs/HPCs) play a major role in improving wound healing is not clear. A small fraction of MSCs present in cord blood may predominantly participate in wound healing. At the very least, UCB likely possesses good potential to repair vascular networks [78]. Because cell culture systems (i.e., serum-free culture) have been relatively well established to comply with FDA regulations, UCB cells are readily available for potential use in the treatment of traumatic injuries and burn.

## 7. Concluding Remarks

Although engraftment of UCB cells and clinical outcomes after UCB transplantation are promising, several aspects of this approach must still be improved. First, mortality due to infection (mostly bacteria) is nearly 50%, and this has yet to be improved much for either BM or UCB transplantation. Second, UCB might have slightly higher risk in terms of relapse of diseases (i.e., leukemia) in exchange for a lower risk in acute GVHD. Some new strategies (neutrophil coinfusion [47] and genetically modified cytotoxic T cells [49]) have been developed to challenge these life-threatening events (infection and relapse) occurring after transplantation. Moreover, recent studies have demonstrated very promising, exciting data that may be applicable to clinical settings in the near future. For example, Notch signaling ligand [71] and aryl hydrocarbon receptor antagonists [73] have appeared as new tools that can be used to establish a better and simpler *ex vivo* cell culture (Table 1). These supplements may change currently used cytokines or feeder cell-based culture systems; however, replacement of cytokine cocktails with these new tools will likely take a while. On the other hand, large-scale culture systems probably need further improvement. Progressive incorporation of cutting-edge techniques in the bioengineering field would be helpful for developing superior culturing devices. Highly efficient large-scale culture hold promise in making UCB an alternative to BM. With well-defined *ex vivo* expansion protocols, UCB is likely to “rock” the area of blood cell transplantation.

## Figures and Tables

**Table 1:** Summary of culture supplements used for expansion.

Supplements	Advantages	Disadvantages
Flt3/Flk2 ligand	Popularly used in numerous studies; serum-free conditions available	Expensive for large-scale culture; difficult to determine the molecular mechanisms of the action; too many combinations, which are not standardized; some ILs may not be necessary
Stem cell factor		
ILs		
Thrombopoietin		
Feeder cell	No need to supply expensive growth factors	Complicated culture systems
Notch ligand	Effective; signaling pathway is relatively clear	Immobilization required; could induce apoptosis under certain conditions
Aryl hydrocarbon receptor antagonists	Defined chemicals, can reduce the cost	Very new, further studies needed
